# A Study on the Relationship of Fat Content in Human Milk on Carotenoids Content and Fatty Acid Compositions in Korea

**DOI:** 10.3390/nu11092072

**Published:** 2019-09-03

**Authors:** Beibei Duan, Jung-Ah Shin, Yan Qin, Jung-Il Kwon, Ki-Teak Lee

**Affiliations:** 1 Department of Food Science and Technology, Chungnam National University, 99 Daehak-ro, Yuseong-gu, Daejeon 34134, Korea; 2 Department of Food Processing and Distribution, Gangneung-Wonju National University, 7 Jukheon-gil, Gangneung, Gangwon-Do 25457, Korea; 3 Maeil Human Milk R&D Center, 63 Jinwiseo-ro, Jinwi-myeon, Pyeongtaek-si, Gyeonggi-do 17714, Korea

**Keywords:** human milk, carotenoids, fatty acid, milk fat

## Abstract

Thirty-four samples of human milk (HM) collected from mothers in Korea were classified into three groups according to their fat content. The lutein + zeaxanthin, β-cryptoxanthin, β-carotene, lycopene, α-carotene, and fatty acids (FAs) present in the HM were quantitatively analyzed. Lutein + zeaxanthin and β-cryptoxanthin were the most abundant carotenoid components in this study, followed by β-carotene. When the classified groups were compared in terms of the content of each carotenoid, there was no statistical difference (*p* > 0.05), indicating that there is no correlation between the content of fat and carotenoid in HM. The mean content of saturated FAs (SFAs), monounsaturated FAs, and polyunsaturated FAs in the analyzed HM were 1.46, 1.36, and 0.83 g/100 g, respectively. In addition, the mean content of arachidonic acid (AA) and docosahexaenoic acid (DHA) were 0.02 and 0.029 g/100 g, respectively. Alternatively, all FAs except for certain SFAs (i.e., C8:0, C10:0, C12:0, and C14:0) did not show statistical difference in composition among the three groups (*p* > 0.05), indicating that the differences in the fat content of HM have limited influence on the FA composition of HM.

## 1. Introduction

Human milk (HM) is the source of various macro- and micro-nutrients for infants, providing the different nutritional needs during infancy stages and building resistance to diseases [1]. Previous studies have reported that the content of nutrients in HM gradually change during the lactation period, in which carotenoids [2,3] and fatty acids (FAs) [4,5,6] were considered for study.

Lutein, zeaxanthin, α/β-cryptoxanthin, lycopene, α/β/ζ-carotene, and phytofluene are the main compositional carotenoids in HM [7]. Carotenoids are usually produced in plants and algae, whereas humans cannot synthesize them. Therefore, lactating mothers need to ingest rich sources of carotenoids such as vegetables and fruits. For example, lycopene is mostly found in tomatoes, while citrus fruits are the best source of β-cryptoxanthin [8]. 

Carotenoids are known to have many physiological functions [9]. For example, carotenoids are the main source of vitamin A and have anti-oxidation, immune regulation, anti-cancer, and anti-aging properties [10]. In particular, α-carotene, β-carotene, and β-cryptoxanthin are known as provitamin A carotenoids and may show some positive effects in humans, such as immune and visual development [11]. Moreover, lutein and zeaxanthin, non-provitamin A carotenoids, prevent eye diseases in particular [12], while lycopene can improve immunity [10]. Therefore, breast-feeding mothers should eat carotenoid-rich foods for infant development and nutrition. 

Until now, a large number of studies have been carried out on the content of carotenoids in mature HM in different countries and regions [2,13,14,15,16,17,18,19], in which lutein + zeaxanthin (19.8–107.6 nmol/L, or 1.1–6.1 μg/100 g), β-cryptoxanthin (12–80 nmol/L), lycopene (14–111.2 nmol/L), α-carotene (3.5–45 nmol/L), and β-carotene (16–88 nmol/L) were reported. However, the carotenoid content in HM seems to be affected by dietary habits [13]. Along with dietary habits, the lactation period also affects the levels of carotenoids in HM. For example, a report on carotenoids in HM samples indicated that the concentrations of carotenoids in colostrum (0–4 days) was significantly higher than that in other lactation periods (5–240 days) except for lutein and zeaxanthin (*p* < 0.01) [15]. 

HM is generally composed of about 3–5% of fat, and more than 98% of milk fat is in the form of triglycerides (TAG), which is an ester molecule derived from glycerol and three fatty acids (FAs) [20]. In particular, the *n*-3 series and *n*-6 series of long-chain polyunsaturated fatty acids (PUFAs) in HM, such as arachidonic acid (AA) and docosahexaenoic acid (DHA), have been considered for owing to their beneficial health effects in early life [21]. Meanwhile, there are several reports on the FA composition in HM samples from different countries [4,5,6,22]. For example, Yuhas et al. [22] reported that the compositional range of saturated fatty acid (SFA), monosaturated fatty acid (MUFA), and PUFA in HM were 35.13–57.06%, 30.33–43.9%, and 11.18–22.33%, respectively, from mothers in nine countries (lactation period: 1–12 months). Among PUFAs, linoleic acid (LA) and α-linolenic acid (ALA) may be affected by the dietary intake because they could not be synthesized de novo by humans (i.e., essential FAs) and must be supplied in the diet [23]. In addition, the content of eicosapentaenoic acid (EPA) and DHA in HM from Japanese mothers were usually higher than those in HM from mothers of other countries owing to the high consumption of fish by mothers in Japan [22]. These results suggest that the content of certain FAs in HM is affected by their dietary habits. 

Carotenoids are classified into xanthophylls (lutein, zeaxanthin, and β-cryptoxanthin) and carotenes (α-carotene, β-carotene, and lycopene) according to their different structures. The xanthophyll molecules contain an oxygen atom, whereas carotenes are composed of carbon and hydrogen. Hence, xanthophylls have higher polarity than carotenes. Nevertheless, carotenoids are considered as lipophilic compounds and they co-exist with fat in the fat droplets dispersed in HM. After the absorption of ingested food, carotenoids are transported by lipoproteins and finally transferred from the blood serum into the mammary glands where milk fat is accumulated and converted into droplets with milk fat globule membranes [3]. 

As there are few results that may help us determine whether HM with high fat content contains more carotenoids, we systematically approached this issue by determining whether there is a difference in carotenoid content according to the fat content of HM. For this purpose, HM from mothers in Korea was classified according to its fat content, and the content ranges (μg/100 g of HM) of the main carotenoids (i.e., lutein + zeaxanthin, β-cryptoxanthin, lycopene, α-carotene, and β-carotene) were obtained. From the results, the correlations between the fat content and individual carotenoid content were studied. In addition, the content (g/100 g of HM) and composition (% of total FA) of FA were obtained to study the correlations between each PUFA and between the fat content and FA composition.

## 2. Materials and Methods

### 2.1. Chemicals and Reagents

Potassium hydroxide, sodium chloride, hexane, ethyl acetate, chloroform, hydrogen chloride, diethyl ether, petroleum ether, and butylated hydroxytoluene (BHT) were purchased from Daejung Chemicals & Metals Co., Ltd. (Shiheung, Korea). Anhydrous sodium sulfate was purchased from Junsei Chemical Co., Ltd. (Tokyo, Japan). Triundecanoin (C11:0, internal standard for FA analysis) was obtained from Nu-Chek Prep, Inc. (Elysian, MN, USA). The solvents (i.e., acetonitrile, methylene chloride, hexane, methyl alcohol, iso-octane, and chloroform) used for high-performance liquid chromatography (HPLC) and gas chromatography (GC) analysis were acquired from Fisher Scientific Korea (Seoul, Korea). BF_3_-methanol (14%, *w*/*w*), pyrogallol, and analytical standards of FA methyl esters (Supelco 37 components FAME mix), lutein (07168-1MG), zeaxanthin (14681-1MG-F), β-cryptoxanthin (C6368-1MG), lycopene (75051-10MG), α-carotene (50887-1MG), and β-carotene (C4582-10MG) were purchased from Sigma-Aldrich Korea (Seoul, Korea). 

### 2.2. Study Design

The study design was approved by the Institutional Review Boards at Maeil Human Milk R&D Center (0627–201306–HRBR–001–02) and Chungnam National University (201808–BR–125–01). All the participants agreed to this study. All HM analyzed in this study were mature milk (42–264 days). The HM samples were loaded into MilkoScan FT2, and then the fat values were obtained in the Maeil Human Milk R&D Center. The samples were sent to our lab in a dry ice packed container, and placed immediately at –20 °C upon arrival. 

**Figure 1:**
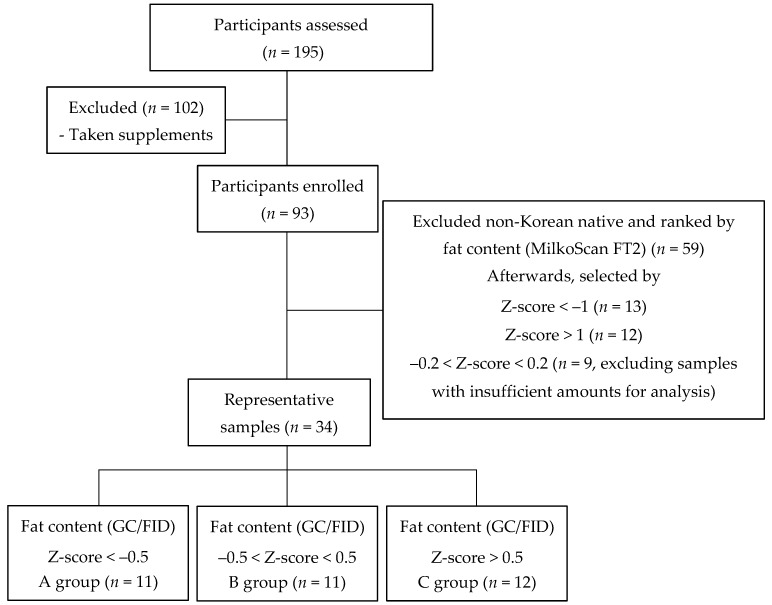
Study flow chart of sample collection.

In this study, the content of carotenoids and FA were analyzed in 34 HM samples from Korean mothers. The screening flowchart is shown in Figure 1. First, a total of 195 participants were assessed for this study. Then, the participants (*n* = 102) who had been taking any supplements were excluded. Afterwards, according to the fat content (from MilkoScan FT2), a representative sample (n = 34) was chosen using a selection process: Z-score < –1 (*n* = 13), Z-score > 1 (*n* = 12), and –0.2 < Z-score < 0.2 (*n* = 9, excluding samples with insufficient amounts for analysis). Finally, according to the fat content (sum of the content of all fatty acids) obtained from GC/FID analysis, a representative sample (*n* = 34) was further classified into three groups using a selection process: Z-score < –0.5 (A group, *n* = 11), –0.5 < Z-score < 0.5 (B group, *n* = 11), and Z-score > 0.5 (C group, *n* = 12). 

### 2.3. Carotenoids Extraction from HM

Carotenoid extraction was done according to the National Laboratory System (NLS) procedure [24] with slight modifications. Aluminum foil was used to package the samples and prevent the degradation of carotenoids in transportation because carotenoids are sensitive to light. HM samples (5 g) were homogenized and weighed, and 10 mL of pyrogallol solution (6% in ethanol) was added into the 200 mL extraction tubes then vortexed for 2 min and flushed with nitrogen for 1 min. After sonication for 10 min, 8 mL of potassium hydroxide solution (60%) was added. Afterwards, the samples were vigorously vortexed (2 min) and placed in a shaking water bath at 75 °C for 1 h at 100 rpm. After incubation, 20 mL of 2% sodium chloride solution was added and 15 mL of extraction solvent (hexane/ethyl acetate, 85/15, *v/v*, 0.01% BHT) was added and vortexed (2 min) to combine organic extracts. After phase separation, the organic layer was transferred to a new volumetric flask (50 mL) through an anhydrous sodium sulfate column. The remaining low layer was extracted twice again with 15 mL extraction solvent. The volumetric flask was filled up with an extraction solvent. Thirty milliliters of combined organic extracts were taken and evaporated under nitrogen to dryness, and then the residue was re-suspended in 1 mL chloroform (HPLC grade), vortexed, and filtered with a 0.5 μm PTFE disposable syringe filter (hydrophobic, Advantec, Tokyo, Japan).

### 2.4. Quantification Analysis of Carotenoids

To separate carotenoids, an HPLC method on a Shiseido Capcell Pak UG120 C18 column (5 μm, 250 × 4.6 mm i.d., Shiseido, Tokyo, Japan) and equipped with an ultraviolet (UV)/Visible detector (set at 450 nm) was used. The solvent system consists of solvent A (acetonitrile/methanol/methylene chloride, 70/10/30, *v*/*v*/*v*) and solvent B (acetonitrile/methyl alcohol/methylene chloride 75/20/5, *v*/*v*/*v*). The gradient elution started at 100% solvent B for 3.5 min and changed to 100% solvent A in 18.5 min, held for 6.5 min, and lastly, changed to 100% solvent B with 1.5 min and held for 10 min. The injection volume was 20 μL and the total run time was 40 min [24].

In order to quantify carotenoids in HM, the stock solutions of the standard should be prepared first. Stock solutions (100 ppm) of lutein and zeaxanthin were individually prepared by dissolving 1 mg of standard in 10 mL of methylene chloride/methyl alcohol (1/1, *v*/*v*). A stock solution of β-cryptoxanthin was prepared by dissolving 1 mg of standard in 10 mL of methylene chloride/methyl alcohol (1/1, *v*/*v*). A stock solution of α-carotene was prepared by dissolving 1 mg of the standard into 2 mL of methylene chloride/methyl alcohol (1/1, *v*/*v*), 1 mL of chloroform (HPLC grade), and 7 mL of hexane to a final concentration of 1 mg/10 mL. A β-carotene standard of 1 mg was dissolved in solvent mixtures of 1 mL of methylene chloride/methyl alcohol (1/1, *v*/*v*), 3 mL of chloroform (HPLC grade), and 6 mL of hexane. A stock solution of lycopene was prepared by dissolving 1 mg of standard in 10 mL of hexane/ethyl acetate (50/50, *v*/*v*). All stock solutions containing 0.01% BHT were freshly prepared before analysis.

The contents of lutein + zeaxanthin, β-cryptoxanthin, lycopene, α-carotene, and β-carotene were quantified with the calibration curves obtained with seven concentrations (0.1, 0.5, 1, 5, 10, 20, and 40 μg/mL). Depending on the concentration of the analyzed sample, each calibration range was obtained for plotting calibration curves as follows: lutein + zeaxanthin, Y = 257.73X − 5.8444 (R^2^ = 0.9966); β-cryptoxanthin, Y = 217X − 5.2638 (R^2^ = 0.9991); lycopene, Y = 141.14X − 0.025 (R^2^ = 1); α-carotene, Y = 267.5X + 0.0749 (R^2^ = 0.9993); and β-carotene, Y = 247.24X − 2.1929 (R^2^ = 1).

### 2.5. Quantification Analysis of FA

Briefly, 1 g of HM sample, 2 mL of pyrogallol solution (50 mg/mL in ethanol solution), 1 mL of triundecanoin (C11:0, 5 mg/mL in iso-octane) as an internal standard, and 10 mL of hydrogen chloride solution (8.3 M) were sequentially added into a 50 mL vial that has a crew-cap. The vial was placed in a shaking water bath at 80 °C for 1 h at 220 rpm after being vortexed for 30 s. Then, 15 mL extraction solvent of diethyl ether was added to the vials while the temperature of vials was lowered in the cold water. The vials were then vortexed for 1 min and centrifugated at 2500 rpm for 3 min, and the supernatant was transferred to new vials through an anhydrous sodium sulfate column. Afterwards, 15 mL of petroleum ether was added to the vial for extraction and then vortexed for 1 min and centrifugation at 2500 rpm for 3 min. All supernatants were then combined into a new extraction vial. The extraction vial was placed in a water bath at 40 °C and flushed with nitrogen until it was completely dried. The content of crude fat was then calculated. For methylation, 2 mL of sodium hydroxide (0.5 N in methanol) was added to each vial, which were vortexed for 30 s and then placed in a water bath at 85 °C for 10 min without shaking. Afterwards, 3 mL of BF_3_-methanol (14%, *w/w*) was added and vortexed for 30 s and left in a water bath at 85 °C for 10 min without shaking. An extraction solvent of iso-octane (3 mL) and saturated sodium chloride solution (1 mL) was added, followed by vortexing for 1 min and centrifuging at 2500 rpm for 3 min. The supernatant was collected for GC/FID analysis through an anhydrous sodium sulfate column [25,26].

FAs were separated with a Agilent 6890 Gas Chromatograph (Santa Clara, USA) fitted with a flame ionization detector. A SP^TM^-2560 capillary column (biscyanopropyl polysiloxane, 100 m × 0.25 mm, 0.25 μm film thickness, Supelco, Bellefonte, PA, USA) was used. Helium was used as a carrier gas and at a flow rate of 1.0 mL/min, and the split ratio was 200:1. The detector and the injector temperatures were set at 285 °C and 225 °C, respectively. One microliter of the sample was injected into the injector port, and the analysis time was for 70 min. The concentration of each FA was calculated with the internal standard, and the results are shown as the content (g/100 g) and composition (% of total FA).

### 2.6. Statistical Analysis

The Shapiro–Wilk test was used to explore whether the content of each analyzed component had a normal distribution. If the data had a normal distribution, a one-way analysis of variance (ANOVA) followed by Duncan’s multiple range test was used to analyze the differences between A, B, and C groups (explained in Figure 1). If not, the differences between groups were compared using a non-parametric Kruskal–Wallis test, and thereafter, the non-parametric Mann–Whitney U test was further used at *p* < 0.05. The correlations between PUFAs as well as between fat content and each carotenoid content were investigated using the Pearman’s rank correlations coefficient. All of the analysis was carried out using the Statistical Package for the Social Sciences (SPSS Inc., Chicago, IL, USA) and the *R* package psych (R Foundation for Statistical Computing, Vienna, Austria). 

## 3. Results 

### 3.1. Carotenoids in HM 

The concentrations of each carotenoid in HM from mothers in Korea are shown in Table 1. The concentrations of lutein and zeaxanthin are expressed as a sum in this study. As shown in Table 1, the amount of total carotenoids (i.e., sum of lutein, zeaxanthin, β-cryptoxanthin, lycopene, α-carotene, and β-carotene) contained in 34 HM samples ranged from 2.68 to 24.89 μg/100 g, suggesting that the concentration of carotenoids was considerably different in individual HM samples. This is also true for the individual components showing wide concentration ranges (e.g., lutein + zeaxanthin (1.15–9.68 μg/100 g), β-cryptoxanthin (0.53–10.96 μg/100 g), and β-carotene (0.49–9.46 μg/100 g), etc.). The mean concentration (μg/100 g) of the carotenoids was the most in lutein + zeaxanthin (3.85) followed by β-cryptoxanthin (3.60); these three components constitute about 73% of the total carotenoids in the analyzed HM. In the case of lycopene (0.29 μg/100 g), it was less than that of β-carotene (1.68 μg/100 g). Among the analyzed carotenoids, the concentration of α-carotene was the lowest (0.19 μg/100 g), being more than eight times lower than that of β-carotene.

In Table 1, provitamin A carotenoids (i.e., the sum of β-cryptoxanthin, β-carotene, and α-carotene) accounted for 53.5% of the total carotenoids. As the content of each carotenoid in the 34 HM samples was widely distributed, the result was shown using medians as well. The highest median (μg/100 g) was observed from β-cryptoxanthin (3.31), in the order of β-cryptoxanthin > lutein + zeaxanthin (3.20) > β-carotene (1.12) > lycopene (0.17) > α-carotene (0.13). Considering that the difference between the mean value of lutein + zeaxanthin (the sum of two components) and β-cryptoxanthin was very small, the median of β-cryptoxanthin was higher than that of lutein + zeaxanthin, and the analyzed six carotenoids generally constitute about 90% of the HM carotenoids [27], β-cryptoxanthin might be one of the most abundant carotenoid components in the Korean HM analyzed in this study. Furthermore, when the content of each carotenoid in the different groups of fat content (i.e., A, B, and C in Table 1 and Figure 1) was examined, there was no statistical difference in the content of any carotenoid among these three groups (*p* > 0.05). However, when we presented the content of each carotenoid per normalized gram of fat (one gram of fat content) contained in HM (Table 2), the content of total carotenoids in HM among these three groups showed a significant difference (*p* < 0.05). Similarly, significant differences between groups were found in the content of lutein + zeaxanthin, β-cryptoxanthin, and β-carotene (*p* < 0.05). On the contrary, lycopene and α-carotene did not exhibit any significant difference (*p* > 0.05), even though the lowest values were found in group C.

### 3.2. Correlations between Fat Content and Carotenoid Content in HM

**Figure 2:**
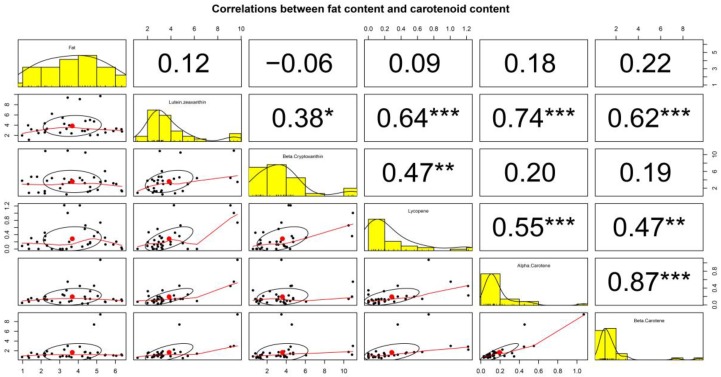
The correlations between fat content and carotenoid content in human milk (HM) from mothers in Korea are graphically shown. The x-axis in each scatter plot represents the column variable, the y-axis the row variable. A histogram of values is represented on the diagonal. The points in the middle of the ellipse below the diagonal represent the points determined by the mean values of the x-axis and y-axis variables. The correlation between two variables is represented by the shape of the ellipse; the more the ellipse is stretched, the stronger its correlation. The curve drawn by the scatter plot represents the general relationship between the x-axis and y-axis variables. The number at the top of the diagonal represents the Pearson correlation coefficient between the x-axis and y-axis variables. *, **, and *** represents significant correlations at *p* < 0.05, *p* < 0.01, and *p* < 0.001, respectively.

To further understand the correlation between the parameters, Pearman’s rank correlations coefficient (*r*) was used (Figure 2). The correlation with the range of −0.06 to 0.22 (*p* > 0.05) was observed, indicating that there is no statistically significant correlation between the fat content and each carotenoid content in HM. With regard to the correlation between each carotenoid content, the strongest correlation was found between α-carotene and β-carotene (*r* = 0.87; *p* < 0.001). Interestingly, lutein + zeaxanthin was significantly correlated with all other carotenoids (*p* < 0.05 and *p* < 0.001). However, no significant correlation was found between β-cryptoxanthin and α-carotene (*r* = 0.20; *p* > 0.05) or between β-cryptoxanthin and β-carotene (*r* = 0.19; *p* > 0.05). 

### 3.3. FA Concentration in HM 

The concentration (g/100 g of HM) and percentage (% of total FA) of FA in HM samples are shown in Table 3 and Table 4, respectively. Palmitic acid (C16:0) was the most abundant SFA, showing a mean content of 0.792 g/100 g. The concentration of C16:0 was in the range of 0.217–1.568 g/100 g (Table 3), which accounted for about 21.38% of total FA (Table 4). The mean content of lauric acid (C12:0), myristic acid (C14:0), and stearic acid (C18:0) was similar (0.199, 0.201, and 0.218 g/100 g, respectively). As a result, the content of total SFA was 1.46 g in 100 g of HM, which accounted for 40.20% of total FA (Table 3 and Table 4). Among the MUFAs, the content of oleic acid (C18:1 *n*-9) was in the range of 0.318–2.093 g/100 g, and it was the predominant FA, with a mean content of 1.168 g/100 g, making about 31.61% of the total FA composition.

In the case of PUFAs, LA (C18:2 *n*-6) showed the highest concentration (0.661 g/100 g) followed by 0.075 g/100 g of ALA (C18:3 *n*-3). The mean ratio of LA to ALA was 10.50 in the wide range of 1.95–24.36. The mean concentrations of AA (C20:4 *n*-6) and DHA (C22:6 *n*-3) were 0.020 and 0.029 g/100 g, respectively, with a ratio (AA/DHA) of 0.93. In addition, the concentration of EPA (C20:5 *n*-3) was 0.009 g/100 g, which was similar to docosapentaenoic acid (C22:5 *n*-3, DPA) content (0.011 g/100 g). Therefore, the ratios of *n*-6/*n*-3 and EPA/DHA were 6.45 and 0.29, respectively (Table 3). Meanwhile, trans FA isomers (i.e., C18:1t, C18:2t, and C18:3t) were found with concentrations of less than 0.03 g/100 g. 

As the groups (i.e., A, B, and C) were classified on the basis of fat content in HM (Figure 1), each FA concentration should be in order of C > B > A. All FAs showed their contents in this order, and the difference was statistically significant between all groups (*p* < 0.05), except for FAs with very low mean content. Furthermore, we investigated whether the difference in fat content affects the FA composition of HM (Table 4). All FA compositions (%), except for C8:0, C10:0, C12:0, and C14:0, and total SFA, did not exhibit any statistical difference (*p* > 0.05) between the three groups, and the maximum difference in the FA composition between the groups was 4.65%, as observed from the total SFA. Therefore, in this study, the difference in the fat content of HM seems to have a limited influence on the FA composition.

### 3.4. Correlations between PUFAs in HM

**Figure 3:**
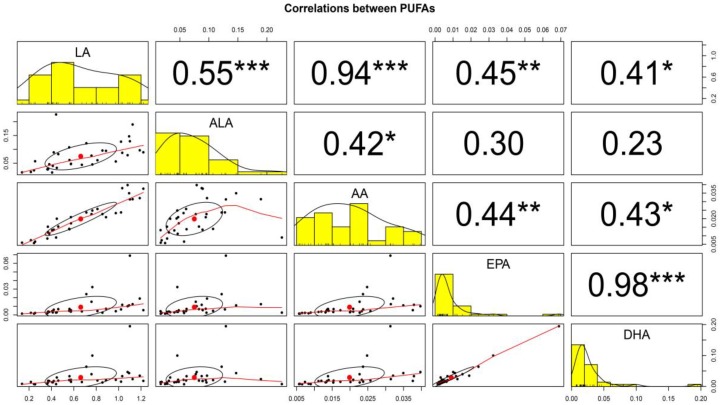
The correlations between polyunsaturated fatty acids (PUFA) contents in HM from mothers in Korea are graphically shown. The x-axis in each scatter plot represents the column variable, the y-axis the row variable. A histogram of values is represented on the diagonal. The points in the middle of the ellipse below the diagonal represent the points determined by the mean values of the x-axis and y-axis variables. The correlation between two variables is represented by the shape of the ellipse; the more the ellipse is stretched, the stronger its correlation. The curve drawn by the scatter plot represents the general relationship between the x-axis and y-axis variables. The number at the top of the diagonal represents the Pearson correlation coefficient between the x-axis and y-axis variables. *, **, and *** represents significant correlations at *p* < 0.05, *p* < 0.01, and *p* < 0.001, respectively. LA, Linoleic acid; ALA, Linolenic acid; AA, Arachidonic acid; EPA, Eicosapentaenoic acid; DHA, Docosahexaenoic acid.

The correlations (r) between PUFAs (i.e., LA, ALA, AA, EPA, and DHA) were also investigated in the 34 HM samples (Figure 3). The correlation between EPA and DHA (*r* = 0.98; *p* < 0.001) was the strongest among the five PUFAs. Furthermore, a strong correlation between LA and AA (*r* = 0.94; *p* < 0.001) in HM was observed. In addition, LA and AA were significantly correlated with all other PUFAs (i.e., ALA, EPA, and DHA) in the range of 0.41–0.55 (*p* < 0.05, *p* < 0.01, and *p* < 0.001) and 0.42–0.44 (*p* < 0.05, and *p* < 0.01), respectively. In contrast, there was no significant correlation between ALA and EPA (*r* = 0.30; *p* > 0.05), and between ALA and DHA (*r* = 0.23; *p* > 0.05).

## 4. Discussion

HM samples from mothers in Korea were selected according to their fat content, and the carotenoid contents were measured. Median (5.32) and mean (5.37) fat content (g/100 g of HM) of group C were 2.8 times more than those of group A, while the content of total carotenoids was not significantly different among the three groups (*p* > 0.05) (Table 1). This result suggests that the fat content may not be related to the carotenoid content in mature HM. As for the mean concentration, lutein + zeaxanthin were the most prominent carotenoids in this study, which is in accordance with previous reports [2,3,15,17,19]. However, in other cases, β-carotene was the most prominent carotenoid [14,18], suggesting that the carotenoid content in HM is related to the dietary habit [13]. Interestingly, β-cryptoxanthin was also predominant, and its content (3.60 μg/100 g) was higher than β-carotene content (1.68 μg/100 g) in this study; similar trends have been found in mature HM from Japan [13] and China (lactation stage: 121–240 days) [15]. However, in the HM from some other countries (e.g., Australia, Canada, Chile, United Kingdom, United States, Germany, and the Philippines), β-carotene (1.18–4.72 μg/100 g) was generally richer than β-cryptoxanthin (0.66–3.35 μg/100 g) [2,13,14,18,19]. The most likely explanation for this difference is that the breast-feeding mothers in north-eastern Asian countries consumed β-cryptoxanthin-rich source of vegetables, fruits, and other food products with preference; for example, β-cryptoxanthin is rich in oranges and tangerines, etc. [8]. As for the content of β-carotene in Korean HM samples, the mean (1.68 μg/100 g) or median (1.12 μg/100 g) was appropriate and within the previously reported ranges (0.86–4.72 μg/100 g) [2,13,14,15,16,17,18,19,28], while the least content was observed from α-carotene among the analyzed carotenoids. The smallest amount of α-carotene in HM has been reported in several studies [3,18,19,28]. Moreover, a previous study has indicated that the provitamin A carotenoids (i.e., α-carotene, β-carotene, and β-cryptoxanthin) accounted for about 59% of total carotenoids measured in HM from nine countries [13], which is in agreement with our findings (53.5%).

With regard to the lycopene content in HM from Korean mothers, the mean (0.29 μg/100 g) and median (0.17 μg/100 g) were much lower than those reported in previous reports from Poland (5.97 μg/100 g) [16], Germany (3.21 μg/100 g) [2], Japan (1.23 μg/100 g) [13], China (1.4–1.5 μg/100 g, lactation stage: 31–240 days) [15], Brazil (0.86 μg/100 g) [17], and the United States (1.18 μg/100 g) [13]. As for the composition of HM, for example, lycopene consisted of 7.1–26.4% of the total carotenoids [2,3,13,14,15,17,18,19,28] in contrast to only 2.8% observed in this study. Therefore, breast-feeding mothers in Korea are assumed to eat less lycopene-rich foods, such as tomatoes, compared with those in other countries. 

The correlation between the content of each carotenoid (i.e., lutein + zeaxanthin, β-cryptoxanthin, lycopene, α-carotene, and β-carotene) and fat content in HM was also observed. In this study, fat content had a correlation coefficient (*r*) of 0.22 with β-carotene and even lower values with other carotenoids. Therefore, the results from Table 1 and Figure 2 suggest that the fat content was not related to the carotenoid content in HM. However, unlike carotenoids, previous studies have shown that the concentration of some fat-soluble vitamins (e.g., retinol and tocopherol) in HM was positively correlated with milk fat content [29,30,31], which has been found in our on-going study. Such discrepancies will need to be studied in the future. 

Although there was no significant difference in the carotenoid content between A, B, and C groups per 100 g of HM, the contents of lutein + zeaxanthin, β-cryptoxanthin, and β-carotene per normalized gram of fat were significantly low in group C. Furthermore, α-carotene and lycopene, which showed relatively low contents, had the lowest content in group C, although there were no significant differences among the three groups (*p* > 0.05). The results suggest that the amount of carotenoids and fat in HM is governed by different mechanisms. While the carotenoid content is closely related to the ingested food absorbed by the mammary epithelial cells, the fat content is affected by the milk-lipid synthesis that intracellularly occurs in the milk-secreting cells of lactating mammary glands; the source of fat is exogenous uptake of FAs and de novo-derived FAs [32]. Furthermore, the rate of transfer from blood plasma to HM after ingestion should be considered. In previous studies, xanthophylls (i.e., lutein, zeaxanthin, and β-cryptoxanthin) have shown greater transfer rates (11.3–53.1%) from blood to breast milk than carotenes (i.e., α-carotene, β-carotene, and lycopene; 1.8–12.2% transfer rates) [2,7]. Therefore, although low intake may be the main reason for the low content of α-carotene, β-carotene, and lycopene in HM from Korean mothers, their low transfer rates may also be affected. In particular, the lycopene content of HM in this study was relatively lower than that of HM from other countries, suggesting that it is desirable for Korean mothers during the feeding period to consume more lycopene-rich foods. Moreover, increasing the lycopene content in infant formula can be an alternative if an infant is to be provided with more lycopene for beneficial functions in the body. 

In addition, a positive correlation between each carotenoid was found in this study, but the strengths of their correlations were different. The highest correlation coefficient (*r*) of 0.87 between α-carotene content and β-carotene content was followed by the order of *r* = 0.74 (between lutein + zeaxanthin and α-carotene) > *r* = 0.64 (between lutein + zeaxanthin and lycopene) > *r* = 0.62 (between lutein + zeaxanthin and β-carotene) > *r* = 0.55 (between α-carotene and lycopene), with a significance at *p* < 0.001 in this study. The highest correlation coefficient (*r* = 0.87) may be due to the fact that α-carotene and β-carotene have similar chemical structures, and they may co-exist in some fruits and vegetables preferred by breast-feeding mothers and have similar absorption mechanisms.

Content (g/100 g) and composition of FAs (% of total FA) in Korean HM was also explored in the present study. Generally, the major compositional TAG molecule in HM is 1,3-dioleoyl-2-palmitoylglycerol, in which palmitic acid (C16:0) is located at the sn-2 position while oleic acid (C18:1 *n*-9) is presented at the sn-1,3 position of TAG molecules. Therefore, oleic acid (21.9–36.4%) and palmitic acid (17.3–24.2%), as the major component FAs, were observed, followed by 7.9–24.3% LA, 4.9–13.2% myristic acid (C14:0), and 4.5–9.8% stearic acid (C18:0). In addition, small amounts of medium-chain FAs (i.e., caprylic acid and capric acid) and PUFAs (e.g., AA, EPA, and DHA, etc.) were contained in HM [33]. As aforementioned, the largest mean amount was observed from oleic acid followed by palmitic acid, which account for 31.61% and 21.38% compositions, respectively (Table 4). The median content of oleic acid was also the highest followed by that of palmitic acid (Table 3).

Unlike other biosynthesized FAs, LA and ALA cannot be synthesized in humans, and such essential FAs should be supplied from the diet. Subsequently, ingested ALA is converted into C20:4 (*n*-3), EPA, and DHA by several elongases and desaturases in the human body while EPA and DHA can also be directly supplied from foods such as fish and algae. Meanwhile, soybean oil and corn oil, which are widely used as cooking oils in Asian countries including China and Korea [23,34], are rich in LA. Within the human body, LA is converted to gamma-linolenic acid (C18:3 *n*-6) by delta-6 desaturase, and AA is subsequently biosynthesized by elongase and delta-5 desaturase, even though AA may be supplied from foods as well. For this reason, it is plausible that the content of some FAs (especially PUFA) in HM may vary depending on the individual diet habit of breast-feeding mothers.

When compared with the previous studies from multiple countries [22], the SFA (40.20%) and MUFA (36.63%) composition in this study was within the expected ranges, i.e., 35.13–57.06% of SFA and 30.33–43.9% of MUFA have been reported. The ratio of *n*-6 PUFA to *n*-3 PUFA was 6.45 with a range of 1.91–11.06 and a median value of 6.55. Moreover, the ratio of LA to ALA (10.50) in Table 3 meets the recommendation of 5–15 [35]. As aforementioned, some PUFA compositions in HM are related to the maternal diet, of which DHA particularly corresponds to the intake of DHA [36]. The content of DHA (mean and median, 0.029 and 0.018 g/100 g; range 0.007–0.195 g/100 g) in this study was larger than that determined in most other countries (range 48.8–149.8 mg/L), except for Japan and the Philippines, in which 337.7–365.4 mg/L were found [22]. The DHA composition (0.77%) of the total FA in Table 4 was also high compared to most of the other countries [22,23]. 

Contrary to the DHA concentration in HM, it seems that the content of AA was less affected by maternal diet [37,38]. The content and composition of AA (mean and median, 0.020 g/100 g; % of total FA, 0.54) in this study was not much different to the global mean value (150.7 mg/L), and the values reported in a previous study are 116.4–207.8 mg/L [22] and 0.42–0.65% [23], respectively. While the European Food Safety Agency (EFSA) suggests that a specific ratio or content of AA and DHA is not required [39], Codex [40] recommends that the concentration of AA should be at least similar to the concentration of DHA and that the concentration of EPA should not be higher than that of DHA when manufacturing infant formula. Thus, if these ratios are required for the normal development of infants, these ratios would also be appropriate when present in HM. As seen in Table 3, the mean and median contents of DHA in HM from Korean mothers were greater than those of EPA, whereas the mean and median of AA/DHA are 0.93 and 0.92, respectively. The worldwide AA/DHA is in the range of 0.51 to 3.52 but is higher than 1 in all regions, except Japan (0.51) and the Philippines (0.62), where DHA was found to have high levels in HM [22,23]. As the beneficial health functions of DHA during infant development are known [21] and DHA in HM seems to be more affected by diet than does AA, balancing such ratios as suggested by the Codex [40] may be possible through providing AA-fortified infant formula without changing the dietary habit of mothers.

Although the amount of each FA increases as the fat content in HM increases, the difference in the fat content of HM is somewhat affected by the FA composition in this study. As the groups (i.e., A, B, and C) were classified by fat content in HM (Figure 1), it is true that the concentrations of all FAs are in the order of C > B > A. However, when FA compositions (%) are considered, all FAs except for some SFAs (i.e., C8:0, C10:0, C12:0, and C14:0) did not exhibit any statistical difference (*p* > 0.05) between the three groups (Table 4). A possible explanation is as follows: The fat content of HM is generally determined by the content of SFA and MUFA produced from genetic and physiological factors (i.e., biosynthesis) as well as the diet, and the content of PUFA is greatly influenced by diet. As shown in Table 4, the total PUFA and MUFA compositions (%) in group C with high fat content was relatively higher than the other groups, although no statistically significant difference was observed (*p* > 0.05). As a result, the total SFA composition (%) of group C was lowered with significance (*p* < 0.05). Therefore, it can be suggested that the fat content of HM, which was affected by diet, had a limited influence on the FA composition. 

In addition, the correlation between LA, ALA, AA, EPA, and DHA content in HM was determined (Figure 3). The results showed positive correlations with different strengths, in which the highest correlation coefficient (*r*) of 0.98 between EPA and DHA was followed by the order of *r* = 0.94 (between LA and AA) > *r* = 0.55 (between LA and ALA) with a significance at *p* < 0.001. Such a high correlation is probably due to the following reasons: Dietary LA is converted to AA by the *n*-6 FA biosynthesis pathway while EPA is converted to DHA by the *n*-3 FA biosynthesis pathway. Therefore, it showed high correlation between the content of FAs in the same biosynthetic pathway. Moreover, EPA and DHA could be taken simultaneously through the diet because they are usually present in seafood such as fish. However, the high correlation between ALA of the *n*-3 family and LA of the *n*-6 family, which are essential FAs, have been derived from ingested food, such as soybean oil used as cooking oil, etc.

## 5. Conclusions

To investigate the relationship between fat content and carotenoids content in HM, lutein + zeaxanthin, β-cryptoxanthin, lycopene, α-carotene, and β-carotene were quantitatively analyzed from Korean mothers’ HM classified by fat content. The results in this study showed that there was no correlation between the fat content and the carotenoid contents in HM, indicating that carotenoids and fat in HM originated from different paths. The mean content of the carotenoids was the highest for lutein + zeaxanthin. Taking the median content of each carotenoid into consideration, β-cryptoxanthin was observed to be the most abundant carotenoid component of Korean HM analyzed in this study. Furthermore, when compared to other populations, the content of carotenoids (i.e., lutein + zeaxanthin, β-cryptoxanthin, α-carotene, and β-carotene) in Korean HM was moderate, except lycopene, which was relatively low. In addition, the content and composition of FAs were reported. The difference in the fat content of HM has a limited influence on the FA composition in this study. Moreover, the ratio of *n*-6 PUFA to *n*-3 PUFA, LA to ALA, and AA to DHA was 6.45, 10.50, and 0.93, respectively, and the highest correlation coefficient was observed between EPA and DHA followed by that between LA and AA in the analyzed HM samples. 

## Figures and Tables

**Table 1:** Contents of carotenoids in human milk from mothers in Korea (μg/100 g).

	Total Mature Milk (*n* = 34)			A Group (*n* = 11)			B Group (*n* = 11)			C Group (*n* = 12)		
	Mean	SD ^1^	Median	Mean	SD	Median	Mean	SD	Median	Mean	SD	Median
Lutein + zeaxanthin	3.85 (1.15–9.68)	2.04	3.20	3.20 (1.15–6.22) ^a^	1.33	3.05	4.63 (2.40–9.38) ^a^	2.47	3.97	3.72 (2.02–9.68) ^a^	2.08	3.18
β-Cryptoxanthin	3.60 (0.53–10.96)	2.67	3.31	3.56 (0.78–10.87) ^a^	2.90	3.78	4.57 (0.53–10.96) ^a^	3.25	3.91	2.76 (1.13–6.07) ^a^	1.54	2.64
Lycopene	0.29 (0.00–1.22)	0.34	0.17	0.17 (0.00–0.45) ^a^	0.16	0.13	0.45 (0.00–1.22) ^a^	0.50	0.20	0.24 (0.00–0.74) ^a^	0.21	0.17
α-Carotene	0.19 (0.03–1.07)	0.20	0.13	0.12 (0.05–0.20) ^a^	0.04	0.13	0.22 (0.03–0.55) ^a^	0.17	0.20	0.24 (0.03–1.07) ^a^	0.29	0.13
β-Carotene	1.68 (0.49–9.46)	1.83	1.12	1.17 (0.57–1.89) ^a^	0.41	1.20	1.50 (0.67–3.00) ^a^	0.86	0.93	2.31 (0.49–9.46) ^a^	2.91	1.12
Total carotenoids	10.22 (2.68–24.89)	5.13	8.52	8.77 (2.68–15.68) ^a^	3.47	8.93	11.84 (5.70–24.89) ^a^	6.03	9.29	10.06 (5.81–24.45) ^a^	5.50	8.06
Fat (g/100 g)	3.68 (0.97–6.36)	1.57	3.57	1.84 (0.97–2.58) ^c^	0.51	1.85	3.67 (3.02–4.38) ^b^	0.50	3.51	5.37 (4.52–6.36) ^a^	0.66	5.32

^1^ Standard deviation. Data expressed as mean (ranges), and median. Mean values with a different letter within a row are significantly different from each other at *p* < 0.05.

**Table 2:** Contents of carotenoids per normalized gram of fat contained in human milk from mothers in Korea (μg/g).

	Total Mature Milk (*n* = 34)			A Group (*n* = 11)			B Group (*n* = 11)			C Group (*n* = 12)		
	Mean	SD ^1^	Median	Mean	SD	Median	Mean	SD	Median	Mean	SD	Median
Lutein + zeaxanthin	1.24 (0.41–3.06)	0.72	1.09	1.77 (0.86–3.06) ^a^	0.64	1.66	1.28 (0.66–2.73) ^a^	0.68	1.10	0.71 (0.41–1.87) ^b^	0.42	0.53
β-Cryptoxanthin	1.24 (0.16–4.89)	1.16	0.83	2.04 (0.35–4.89) ^a^	1.51	1.71	1.23 (0.16–3.19) ^a^	0.85	0.97	0.52 (0.20–1.12) ^b^	0.29	0.49
Lycopene	0.09 (0.00–0.38)	0.10	0.05	0.09 (0.00–0.21) ^a^	0.08	0.10	0.12 (0.00–0.38) ^a^	0.14	0.06	0.05 (0.00–0.14) ^a^	0.04	0.03
α-Carotene	0.06 (0.01–0.21)	0.04	0.05	0.07 (0.04–0.11) ^a^	0.02	0.06	0.06 (0.01–0.16) ^a^	0.05	0.04	0.05 (0.01–0.21) ^a^	0.06	0.02
β-Carotene	0.50 (0.11–1.82)	0.39	0.40	0.65 (0.41–1.09) ^a^	0.21	0.67	0.41 (0.19–0.87) ^b^	0.24	0.30	0.45 (0.11–1.82) ^b^	0.58	0.20
Total carotenoids	3.33 (0.91–7.99)	2.02	2.65	4.96 (2.00–7.99) ^a^	2.01	4.70	3.23 (1.69–7.26) ^b^	1.65	2.65	1.92 (0.91–4.71) ^c^	1.12	1.52

^1^ Standard deviation. Data expressed as mean (ranges) and median. Mean values with a different letter within a row are significantly different from each other at *p* < 0.05.

**Table 3:** Contents of fatty acids in human milk from mothers in Korea (g/100 g).

	Total Mature Milk (*n* = 34)			A Group (*n* = 11)			B Group (*n* = 11)			C Group (*n* = 12)		
	Mean	SD ^1^	Median	Mean	SD	Median	Mean	SD	Median	Mean	SD	Median
C8:0	0.003 (0.000–0.007)	0.002	0.003	0.001 (0.000–0.002) ^c^	0.001	0.000	0.003 (0.001–0.004) ^b^	0.001	0.003	0.005 (0.002–0.007) ^a^	0.001	0.005
C10:0	0.045 (0.010–0.079)	0.021	0.045	0.022 (0.010–0.036) ^b^	0.009	0.023	0.051 (0.032–0.068) ^a^	0.010	0.049	0.061 (0.026–0.079) ^a^	0.018	0.068
C12:0	0.199 (0.060–0.379)	0.090	0.197	0.108 (0.060–0.178) ^b^	0.044	0.111	0.238 (0.191–0.292) ^a^	0.040	0.242	0.246 (0.078–0.379) ^a^	0.094	0.259
C14:0	0.201 (0.054–0.342)	0.083	0.206	0.111 (0.054–0.187) ^b^	0.048	0.115	0.240 (0.153–0.311) ^a^	0.049	0.235	0.247 (0.140–0.342) ^a^	0.065	0.239
C16:0	0.792 (0.217–1.568)	0.368	0.774	0.391 (0.217–0.592) ^c^	0.124	0.369	0.789 (0.561–0.957) ^b^	0.126	0.771	1.164 (0.797–1.568) ^a^	0.257	1.124
C18:0	0.218 (0.048–0.385)	0.093	0.223	0.114 (0.048–0.155) ^c^	0.035	0.126	0.225 (0.157–0.349) ^b^	0.051	0.218	0.306 (0.246–0.385) ^a^	0.053	0.289
C20:0	0.006 (0.001–0.011)	0.003	0.006	0.003 (0.001–0.004) ^c^	0.001	0.003	0.006 (0.005–0.009) ^b^	0.001	0.006	0.008 (0.006–0.011) ^a^	0.001	0.008
C14:1	0.006 (0.001–0.016)	0.003	0.005	0.003 (0.001–0.008) ^b^	0.002	0.002	0.006 (0.003–0.012) ^a^	0.003	0.005	0.008 (0.003–0.016) ^a^	0.004	0.007
C16:1	0.094 (0.023–0.200)	0.051	0.088	0.043 (0.023–0.068) ^c^	0.014	0.040	0.090 (0.054–0.126) ^b^	0.027	0.092	0.145 (0.084–0.200) ^a^	0.039	0.142
C18:1 *n*-9	1.168 (0.318–2.093)	0.535	1.149	0.563 (0.318–0.698) ^c^	0.132	0.573	1.144 (0.908–1.384) ^b^	0.172	1.127	1.746 (1.351–2.093) ^a^	0.293	1.792
C18:1 *n*-7	0.069 (0.020–0.124)	0.032	0.066	0.033 (0.020–0.049) ^c^	0.009	0.033	0.069 (0.054–0.091) ^b^	0.012	0.066	0.104 (0.085–0.124) ^a^	0.013	0.107
C18:1t	0.005 (0.001–0.013)	0.002	0.004	0.002 (0.001–0.004) ^c^	0.001	0.002	0.004 (0.002–0.006) ^b^	0.001	0.004	0.07 (0.004–0.013) ^a^	0.002	0.006
C18:2t	0.011 (0.004–0.021)	0.005	0.011	0.006 (0.004–0.013) ^c^	0.003	0.006	0.011 (0.008–0.015) ^b^	0.002	0.011	0.016 (0.009–0.021) ^a^	0.003	0.017
C18:3t	0.009 (0.002–0.068)	0.011	0.007	0.004 (0.002–0.007) ^c^	0.001	0.003	0.008 (0.005–0.012) ^b^	0.002	0.007	0.016 (0.008–0.068) ^a^	0.017	0.010
C20:1	0.017 (0.003–0.116)	0.019	0.013	0.006 (0.003–0.009) ^c^	0.002	0.007	0.015 (0.011–0.024) ^b^	0.004	0.013	0.029 (0.018–0.116) ^a^	0.028	0.021
C18:2 *n*-6 (LA)	0.661 (0.137–1.220)	0.319	0.574	0.331 (0.137–0.442) ^c^	0.101	0.368	0.615 (0.371–1.021) ^b^	0.183	0.573	1.006 (0.758–1.220) ^a^	0.158	1.056
C18:3 *n*-6	0.003 (0.000–0.008)	0.002	0.002	0.001 (0.000–0.002) ^b^	0.001	0.001	0.003 (0.001–0.005) ^a^	0.001	0.003	0.004 (0.002–0.008) ^a^	0.002	0.004
C18:3 *n*-3 (ALA)	0.075 (0.017–0.226)	0.049	0.061	0.054 (0.017–0.226) ^b^	0.061	0.028	0.070 (0.031–0.128) ^a,b^	0.035	0.061	0.099 (0.046–0.190) ^a^	0.040	0.092
C20:2	0.012 (0.003–0.024)	0.006	0.012	0.006 (0.003–0.008) ^c^	0.002	0.007	0.012 (0.008–0.017) ^b^	0.003	0.013	0.017 (0.011–0.024) ^a^	0.004	0.018
C20:3 *n*-6	0.014 (0.003–0.031)	0.007	0.014	0.006 (0.003–0.010) ^c^	0.002	0.006	0.014 (0.008–0.021) ^b^	0.004	0.014	0.021 (0.012–0.031) ^a^	0.006	0.020
C20:4 *n*-6 (AA)	0.020 (0.006–0.040)	0.010	0.020	0.010 (0.006–0.015) ^c^	0.003	0.009	0.019 (0.012–0.026) ^b^	0.005	0.020	0.030 (0.021–0.040) ^a^	0.007	0.031
C20:5 *n*-3 (EPA)	0.009 (0.001–0.069)	0.013	0.005	0.003 (0.001–0.006) ^c^	0.001	0.003	0.007 (0.003–0.024) ^b^	0.006	0.005	0.017 (0.003–0.069) ^a^	0.018	0.011
C22:5 *n*-3 (DPA)	0.011 (0.003–0.034)	0.007	0.009	0.005 (0.003–0.007) ^c^	0.001	0.005	0.010 (0.007–0.021) ^b^	0.004	0.009	0.016 (0.008–0.034) ^a^	0.007	0.015
C22:6 *n*-3 (DHA)	0.029 (0.007–0.195)	0.035	0.018	0.012 (0.007–0.017) ^b^	0.004	0.013	0.024 (0.014–0.064) ^a^	0.014	0.019	0.049 (0.013–0.195) ^a^	0.051	0.034
Total SFA	1.46 (0.40–2.58)	0.60	1.53	0.75 (0.40–1.08) ^c^	0.24	0.68	1.55 (1.24–1.87) ^b^	0.19	1.56	2.04 (1.50–2.58) ^a^	0.36	1.99
Total MUFA	1.36 (0.38–2.42)	0.62	1.37	0.65 (0.38–0.82) ^c^	0.15	0.66	1.32 (1.03–1.60) ^b^	0.19	1.34	2.03 (1.55–2.42) ^a^	0.32	2.11
Total PUFA	0.83 (0.18–1.59)	0.40	0.72	0.43 (0.18–0.71) ^c^	0.15	0.45	0.77 (0.46–1.24) ^b^	0.22	0.71	1.26 (0.97–1.59) ^a^	0.21	1.30
*n*-6	0.70 (0.15–1.29)	0.34	0.61	0.35 (0.15–0.46) ^c^	0.11	0.39	0.65 (0.39–1.07) ^b^	0.19	0.61	1.06 (0.80–1.29) ^a^	0.17	1.13
*n*-3	0.12 (0.03–0.43)	0.08	0.11	0.07 (0.03–0.24) ^b^	0.06	0.05	0.11 (0.06–0.23) ^b^	0.05	0.09	0.18 (0.09–0.43) ^a^	0.09	0.15
LA/ALA	10.50 (1.95–24.36)	4.40	10.08	9.99 (1.95–24.36) ^a^	5.99	9.97	10.17 (5.21–15.17) ^a^	3.56	11.95	11.29 (5.93–17.31) ^a^	3.59	10.79
AA/DHA	0.93 (0.16–2.10)	0.41	0.92	0.87 (0.51–1.20) ^a^	0.22	0.92	0.91 (0.37–1.54) ^a^	0.40	0.82	1.00 (0.16–2.10) ^a^	0.56	0.97
EPA/DHA	0.29 (0.11–0.55)	0.10	0.27	0.25 (0.16–0.35) ^a^	0.07	0.22	0.27 (0.11–0.42) ^a^	0.10	0.23	0.33 (0.20–0.55) ^a^	0.11	0.34
*n*-6/*n*-3	6.45 (1.91–11.06)	2.34	6.55	6.14 (1.91–10.87) ^a^	2.60	5.77	6.48 (3.27–10.04) ^a^	2.15	6.52	6.70 (2.71–11.06) ^a^	2.42	7.06

^1^ Standard deviation. Data expressed as mean (ranges) and median. Mean values with a different letter within a row are significantly different from each other at *p* < 0.05.

**Table 4:** Fatty acid compositions in human milk from mothers in Korea (% of total fatty acids (FA)).

	Total Mature Milk (*n* = 34)			A Group (*n* = 11)			B Group (*n* = 11)			C Group (*n* = 12)		
	Mean	SD^1^	Median	Mean	SD	Median	Mean	SD	Median	Mean	SD	Median
C8:0	0.07 (0.00–0.13)	0.04	0.07	0.03 (0.00–0.08) ^b^	0.04	0.00	0.08 (0.05–0.13) ^a^	0.02	0.07	0.08 (0.04–0.12) ^a^	0.02	0.09
C10:0	1.23 (0.51–1.77)	0.27	1.17	1.16 (0.87–1.55) ^b^	0.23	1.09	1.39 (1.07–1.77) ^a^	0.24	1.40	1.14 (0.51–1.52) ^b^	0.29	1.16
C12:0	5.63 (1.55–8.34)	1.68	5.66	5.87 (3.57–8.14) ^a^	1.55	6.13	6.57 (4.39–8.34) ^a^	1.24	6.92	4.56 (1.55–7.31) ^b^	1.64	4.24
C14:0	5.72 (2.76–8.99)	1.74	5.53	6.00 (2.93–8.51) ^a^	1.73	6.46	6.68 (4.11–8.99) ^a^	1.77	7.04	4.59 (2.76–6.60) ^b^	1.03	4.42
C16:0	21.38 (15.37–24.67)	2.09	21.70	21.16 (17.62–23.94) ^a^	1.73	21.31	21.42 (18.54–22.69) ^a^	1.13	21.71	21.56 (15.37–24.67) ^a^	3.02	22.83
C18:0	6.00 (4.75–9.81)	1.05	5.68	6.20 (4.83–9.81) ^a^	1.37	6.02	6.12 (5.19–8.29) ^a^	1.00	5.64	5.71 (4.75–7.52) ^a^	0.75	5.63
C20:0	0.16 (0.12–0.29)	0.03	0.16	0.17 (0.13–0.29) ^a^	0.04	0.16	0.17 (0.15–0.20) ^a^	0.02	0.16	0.15 (0.12–0.19) ^a^	0.02	0.16
C14:1	0.15 (0.07–0.38)	0.07	0.14	0.15 (0.09–0.32) ^a^	0.07	0.14	0.16 (0.08–0.38) ^a^	0.09	0.15	0.14 (0.07–0.26) ^a^	0.06	0.14
C16:1	2.55 (1.28–3.88)	0.72	2.61	2.43 (1.45–3.73) ^a^	0.70	2.63	2.49 (1.28–3.88) ^a^	0.85	2.31	2.71 (1.81–3.85) ^a^	0.65	2.69
C18:1 *n*-9	31.61 (26.14–39.58)	2.97	31.18	31.11 (26.14–35.53) ^a^	3.05	31.38	31.16 (28.68–35.45) ^a^	2.24	30.24	32.47 (27.84–39.58) ^a^	3.49	31.77
C18:1 *n*-7	1.88 (1.31–2.45)	0.25	1.88	1.80 (1.31–2.23) ^a^	0.29	1.75	1.88 (1.51–2.40) ^a^	0.27	1.87	1.94 (1.72–2.45) ^a^	0.20	1.92
C18:1t	0.12 (0.08–0.21)	0.03	0.12	0.13 (0.11–0.16) ^a^	0.02	0.12	0.12 (0.08–0.18) ^a^	0.03	0.12	0.13 (0.09–0.21) ^a^	0.03	0.11
C18:2t	0.32 (0.18–0.49)	0.06	0.32	0.34 (0.23–0.49) ^a^	0.07	0.32	0.31 (0.22–0.40) ^a^	0.05	0.32	0.30 (0.18–0.37) ^a^	0.05	0.31
C18:3t	0.24 (0.13–1.41)	0.21	0.19	0.19 (0.13–0.32) ^a^	0.05	0.19	0.20 (0.14–0.29) ^a^	0.05	0.20	0.31 (0.15–1.41) ^a^	0.35	0.20
C20:1	0.44 (0.25–2.39)	0.35	0.37	0.35 (0.25–0.44) ^a^	0.05	0.34	0.41 (0.30–0.58) ^a^	0.08	0.37	0.56 (0.33–2.39) ^a^	0.58	0.38
C18:2 *n*-6 (LA)	17.77 (11.43–23.58)	2.73	17.39	17.87 (14.16–20.52) ^a^	1.82	17.55	16.51 (11.43–23.29) ^a^	3.12	16.37	18.84 (14.74–23.58) ^a^	2.77	18.48
C18:3 *n*-6	0.07 (0.00–0.16)	0.04	0.08	0.07 (0.00–0.12) ^a^	0.04	0.08	0.08 (0.04–0.15) ^a^	0.04	0.07	0.08 (0.03–0.16) ^a^	0.04	0.07
C18:3 *n*-3 (ALA)	2.15 (0.72–8.76)	1.47	1.76	2.69 (0.72–8.76) ^a^	2.27	1.82	1.90 (0.95–3.55) ^a^	0.94	1.39	1.87 (0.88–3.17) ^a^	0.74	1.72
C20:2	0.33 (0.22–0.47)	0.06	0.32	0.34 (0.27–0.47) ^a^	0.07	0.30	0.32 (0.25–0.41) ^a^	0.05	0.32	0.32 (0.22–0.43) ^a^	0.06	0.31
C20:3 *n*-6	0.36 (0.17–0.56)	0.08	0.38	0.33 (0.17–0.46) ^a^	0.08	0.33	0.38 (0.23–0.51) ^a^	0.07	0.40	0.38 (0.23–0.56) ^a^	0.09	0.39
C20:4 *n*-6 (AA)	0.54 (0.35–0.75)	0.10	0.55	0.54 (0.35–0.70) ^a^	0.10	0.57	0.51 (0.37–0.59) ^a^	0.07	0.49	0.56 (0.40–0.75) ^a^	0.11	0.54
C20:5 *n*-3 (EPA)	0.22 (0.05–1.42)	0.25	0.16	0.16 (0.10–0.27) ^a^	0.05	0.15	0.18 (0.09–0.59) ^a^	0.14	0.15	0.33 (0.05–1.42) ^a^	0.38	0.20
C22:5 *n*-3 (DPA)	0.28 (0.17–0.69)	0.11	0.27	0.25 (0.17–0.37) ^a^	0.06	0.23	0.27 (0.19–0.51) ^a^	0.09	0.25	0.31 (0.17–0.69) ^a^	0.14	0.30
C22:6 *n*-3 (DHA)	0.77 (0.25–4.01)	0.67	0.59	0.67 (0.29–1.12) ^a^	0.24	0.60	0.67 (0.34–1.56) ^a^	0.36	0.59	0.96 (0.25–4.01) ^a^	1.06	0.59
Total SFA	40.20 (30.85–48.46)	3.72	40.55	40.59 (33.00–46.70) ^a,b^	3.60	41.27	42.44 (37.11–48.46) ^a^	3.18	42.87	37.79 (30.85–40.58) ^b^	3.00	38.86
Total MUFA	36.63 (29.54–44.68)	3.44	36.26	35.85 (29.54–40.68) ^a^	3.64	36.63	36.11 (33.94–41.86) ^a^	2.94	34.82	37.82 (33.34–44.68) ^a^	3.64	36.63
Total PUFA	22.49 (14.19–32.88)	3.79	22.17	22.91 (18.57–27.52) ^a^	2.73	22.87	20.82 (14.19–28.31) ^a^	3.91	19.83	23.64 (19.11–32.88) ^a^	4.23	22.73

^1^ Standard deviation. Data expressed as mean (ranges) and median. Mean values with a different letter within a row are significantly different from each other at *p* < 0.05.
